# Hantavirus preparedness at human–rodent interfaces: lessons from China’s HFRS control and Andes virus transmission in mobile populations

**DOI:** 10.1080/22221751.2026.2721722

**Published:** 2026-09-22

**Authors:** Xinyu Wang, Wenhong Zhang

**Affiliations:** Department of Infectious Diseases, Huashan Hospital, Fudan University, Shanghai, China

**Keywords:** Hantavirus, Andes virus, haemorrhagic fever with renal syndrome, rodent-borne zoonoses, preparedness

## Abstract

Hantaviruses are globally distributed reservoir-associated viruses that cause haemorrhagic fever with renal syndrome (HFRS), mainly in Eurasia, and hantavirus cardiopulmonary syndrome (HCPS; also termed hantavirus pulmonary syndrome, HPS), mainly in the Americas. Most human infections arise from exposure to rodent-contaminated environments, so clustered disease may reflect shared environmental exposure rather than interpersonal transmission. This Review uses China’s long-term HFRS experience as a central case study for interface-centred preparedness. China demonstrates that sustained surveillance, reservoir monitoring, risk-defined vaccination, rodent control and environmental risk reduction can substantially reduce disease burden, but declining incidence does not mean disappearance of ecological risk. Its dual HTNV and SEOV ecology further shows that one clinical syndrome can arise from distinct agricultural and peri-domestic rat-associated interfaces. Andes virus (ANDV) provides a contrasting challenge: selected outbreaks have documented limited but consequential human-to-human transmission after close or prolonged contact. The 2026 *MV Hondius* event illustrates how such transmission can become an international contact-tracing problem when it occurs in mobile populations. Preparedness should therefore combine long-term management of rodent-derived interfaces, rapid discrimination between common-source clusters and ANDV secondary transmission, and response systems that remain effective when exposed persons, infected animals or close contacts cross geographical borders.

## Introduction: why interfaces matter for hantavirus preparedness

Hantaviruses are often discussed as a single group of rodent-borne viruses, but their public health significance depends on the settings in which exposure and transmission occur. For most medically important hantaviruses, human disease follows exposure to environments contaminated by infected rodent excreta, and multiple cases may arise when people share the same agricultural, household, occupational or travel-related exposure. Such clustering does not, by itself, indicate transmission between people. Andes virus (ANDV) represents an important exception: selected outbreaks have demonstrated secondary human-to-human transmission, usually after close or prolonged contact, although this pattern remains distinct from efficient community spread. This interface-based distinction is summarized in Figure 1.

**Figure 1.**
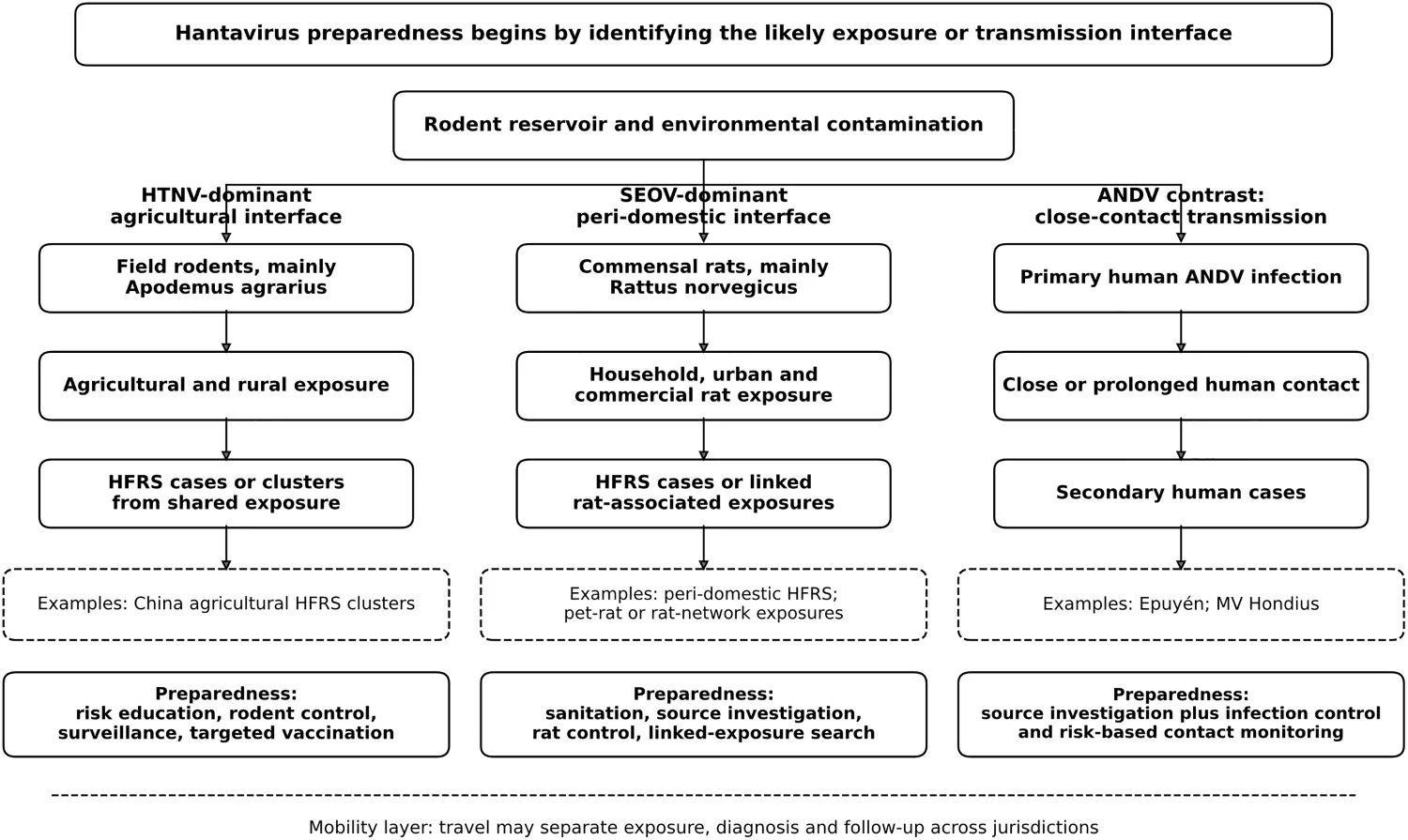
Interface-centred framework for hantavirus preparedness. China’s long-term HFRS experience is represented by two predominant rodent-derived interfaces: an HTNV-dominant agricultural interface and an SEOV-dominant peri-domestic interface. Both may produce clustered cases through shared environmental exposure. ANDV differs because an initial rodent-derived infection may be followed by limited secondary human-to-human transmission after close or prolonged contact. The mobility layer indicates that travel may separate exposure, diagnosis and follow-up across geographical borders. Abbreviations: ANDV, Andes virus; HFRS, haemorrhagic fever with renal syndrome; HTNV, Hantaan virus; SEOV, Seoul virus.

Rather than providing another broad overview of hantavirus taxonomy, clinical disease and countermeasures, this Review asks how preparedness should be organized around the pathways that generate risk. China provides the central case study because it combines the world’s largest documented HFRS experience, long-term national surveillance, co-circulation of HTNV and SEOV, vaccination programmes, and continuing ecological change despite marked declines in reported incidence. The representative virus-reservoir interfaces that anchor this argument are summarized in Table 1.

**Table 1.** Representative hantavirus interfaces and public health implications.

Virus	Reservoir interface	Main human syndrome	Cluster interpretation	Public health implication
HTNV	Field-rodent exposure, mainly involving the striped field mouse (*Apodemus agrarius*) in agricultural and rural settings	HFRS	Usually reflects shared environmental exposure rather than person-to-person transmission	Agricultural risk reduction, rodent monitoring, rural health education, and targeted vaccination where appropriate
SEOV	Peri-domestic and urban rat exposure, mainly involving the Norway rat (*Rattus norvegicus*), including household and commercial settings	HFRS	May involve several exposed persons through shared rat-associated exposure; does not establish sustained person-to-person transmission	Household and commercial rat control, sanitation, food storage protection, and investigation of linked rat-associated exposures
PUUV	Forest and peri-forest exposure involving the bank vole (*Myodes glareolus*)	Usually nephropathia epidemica (milder HFRS)	Clusters usually reflect shared environmental exposure	Exposure avoidance in endemic outdoor settings, clinical recognition, and locally adapted surveillance
DOBV	Exposure involving Apodemus mice in endemic areas	Potentially severe HFRS	Clusters usually reflect shared environmental exposure	Region-specific surveillance, early recognition, and supportive care capacity in affected areas
SNV	Exposure to deer mouse (*Peromyscus maniculatus*)-contaminated cabins, homes or field accommodation	HCPS	Clusters should first be interpreted as common-source environmental events	Environmental investigation, safe cleaning guidance, clinical alerting, and identification of others exposed to the same setting
ANDV	Initial rodent-derived exposure, followed in selected settings by close-contact transmission among humans	HCPS	Requires distinction between a shared environmental source and secondary human-to-human transmission	Environmental source investigation plus close-contact assessment, infection control, and contact monitoring when ANDV infection is suspected or confirmed

Abbreviations: ANDV, Andes virus; DOBV, Dobrava-Belgrade virus; HCPS, hantavirus cardiopulmonary syndrome; HFRS, haemorrhagic fever with renal syndrome; HTNV, Hantaan virus; PUUV, Puumala virus; SEOV, Seoul virus; SNV, Sin Nombre virus.

The 2026 *MV Hondius* ANDV event adds a contrasting public health lesson. It should not be treated as the sole foundation for hantavirus preparedness, because the relative contributions of specific transmission routes remain under investigation. Instead, it illustrates a different operational problem: when limited ANDV secondary transmission occurs in a mobile population, exposure, clinical recognition, contact identification and public health follow-up may be separated across countries. This Review therefore uses China’s long-term HFRS control experience as the central case study for interface-centred preparedness and contrasts it with ANDV transmission in mobile populations. We focus on four linked questions: how reservoir-specific settings generate human risk; what China’s HFRS experience contributes to global preparedness; how common-source environmental clusters differ from ANDV secondary transmission; and which surveillance, diagnostic, preventive and response tools are needed for the next decade.

## Narrative review approach

This is a narrative review. We prioritized peer-reviewed studies and official public health documents that informed four issues: reservoir-specific settings for human hantavirus disease; China’s long-term HFRS surveillance, vaccination and control experience; the distinction between common-source environmental clusters and ANDV secondary transmission; and operational tools for preparedness in endemic and mobile populations. We searched PubMed, Web of Science and official sources from WHO, CDC and China CDC using terms including hantavirus, HFRS, HCPS, HPS, Hantaan virus, Seoul virus, Andes virus, China, surveillance, vaccination, rodent reservoir, climate, land use, urbanization, travel, transmission and preparedness. We prioritized recent evidence where available, while retaining landmark field investigations and outbreak studies when they established key epidemiological or operational principles.

## Hantavirus diversity: why reservoir settings matter

Hantaviruses belong to the family *Hantaviridae* within the order *Bunyavirales*. Medically important rodent-borne hantaviruses are classified mainly within the subfamily *Mammantavirinae* and include orthohantaviruses associated with two major severe human syndromes: haemorrhagic fever with renal syndrome (HFRS), reported predominantly in Eurasia, and hantavirus cardiopulmonary syndrome (HCPS; also termed hantavirus pulmonary syndrome, HPS), reported mainly in the Americas. Their shared genomic organization and endothelial tropism do not translate into uniform epidemiology, clinical risk or preparedness needs.

The practical differences among hantaviruses arise from the combination of reservoir host, exposure setting, dominant clinical syndrome and transmission pathway. HTNV and SEOV are major causes of HFRS in Asia, but they are associated with different reservoir settings: HTNV with field rodents such as A. agrarius, and SEOV with commensal rats such as R. norvegicus. PUUV and DOBV illustrate additional Old World diversity, with PUUV usually causing milder nephropathia epidemica and DOBV causing potentially severe HFRS. In the Americas, SNV and ANDV are major HCPS-associated viruses, characterized clinically by pulmonary capillary leak and circulatory compromise. Receptor use also varies across hantavirus groups: beta-3 integrins have been implicated in cellular entry of pathogenic hantaviruses, decay-accelerating factor (DAF/CD55) has been reported as a cofactor for Hantaan and Puumala virus infection, and protocadherin-1 is an experimentally established entry determinant for several New World HCPS-associated hantaviruses [1–3]. These differences reinforce the need to avoid treating hantavirus disease as a single operational category. Representative hantavirus interfaces and their public health implications are summarized in Table 1.

For preparedness, the key distinction is not only HFRS versus HCPS, but also how infection is acquired. Most human hantavirus infections follow exposure to rodent-contaminated environments. In such settings, several cases may occur within a household, farm, cabin, military camp or travel group because multiple people encountered the same contaminated environment. ANDV adds a second pathway: after an initial rodent-derived infection, secondary transmission may occur among close human contacts. The sections below use China’s HFRS experience to examine persistent rodent-derived risk and ANDV to examine the added challenge of close-contact transmission in mobile populations. Figure 1 presents this two-pathway logic as the conceptual framework for the Review.

## China as a long-term natural experiment in interface-centred HFRS preparedness

### Long surveillance record: decline without disappearance

China provides one of the most informative long-term examples of hantavirus preparedness at reservoir-human interfaces. Between 1950 and 2007, more than 1.5 million HFRS cases and over 46,000 deaths were reported in China, making it historically the country with the largest documented HFRS burden [4]. National surveillance after 2006 showed a continued burden but a declining trend: from 2006 to 2012, 77,558 cases and 866 deaths were reported, with marked geographic heterogeneity across provinces [5]. Analyses of major endemic areas from 2004 to 2016 further indicated that declining incidence coexisted with continuing hantavirus circulation and molecular evolution [6]. More recent surveillance from 2014 to 2023 confirmed that HFRS remains a public health threat: affected areas have expanded, and diverse HTNV and SEOV genotypes continue to circulate in rodent populations [7]. The first lesson from China is therefore that successful reduction in reported human disease should not be equated with ecological elimination. For rodent-borne viruses, the reservoir-virus system may persist, shift and re-emerge even when incidence falls.

Spatial analyses strengthen this point. National and subnational studies have shown persistent clustering, regional heterogeneity and changing hotspots of HFRS in China [8–10]. These studies support a risk-stratified approach to preparedness: surveillance and prevention should be adjusted to where reservoir settings remain active, rather than applied uniformly according to administrative boundaries or historical endemicity alone. China’s experience therefore moves the discussion from whether HFRS is declining to which rodent-human settings continue to generate risk despite overall decline.

### Dual reservoir settings: HTNV and SEOV

A second lesson is that HFRS preparedness cannot be organized around the clinical syndrome alone. China’s experience demonstrates that a single syndrome can arise from distinct reservoir settings requiring different prevention strategies. Early field investigations in Anhui Province showed that HFRS risk was associated with agricultural and rural exposure, including sleeping on the ground or in straw huts, heavy farm work and threshing activities [11]. These findings supported a common-source environmental explanation for clustered disease rather than interpersonal transmission. A subsequent retrospective and prospective study in rural Zhejiang Province identified risk factors linked to both field and household environments, including rat-contaminated food, direct rodent contact, camping in grain fields, straw storage near houses and living on the village periphery [12]. Together with earlier national epidemiological studies distinguishing Apodemus-associated rural disease from Rattus-associated urban disease, these data support a dual-interface model of HFRS in China [13].

Operationally, HTNV maintained by A. agrarius is primarily linked to agricultural and rural field exposure, whereas SEOV maintained by R. norvegicus is linked to household, peri-domestic, urban and commensal-rat exposure. This distinction changes the public health question. HTNV prevention emphasizes agricultural work, crop storage, rural housing, field exposure and seasonal education. SEOV prevention emphasizes household and commercial rat control, food storage, urban sanitation, ports, warehouses, laboratories, breeding facilities and pet-rat networks. Preparedness should therefore distinguish the reservoir setting that generates exposure, rather than treating HFRS as a uniform operational problem. Table 1 makes this difference explicit across representative hantaviruses.

The dual-interface model remains useful but should not be understood as a fixed rural-urban dichotomy. Recent analyses of HTNV and SEOV distribution in China from 2001 to 2020 indicate that urbanization-related factors are associated with changing spatial patterns of Hantaan and Seoul virus infections [14]. This suggests that the classical distinction between agricultural HTNV risk and peri-domestic SEOV risk is being modified by urbanization, mixed land use, peri-urban expansion, housing changes, food storage and occupational patterns. Urbanization does not simply replace rural HFRS risk with urban risk; it can create overlapping settings in which field rodents, commensal rats and human exposure behaviours interact.

### Vaccination as programmatic interface control

China’s vaccination experience provides a third programmatic lesson. HFRS vaccination has been used in China for more than two decades, and since 2008 expanded immunization has been implemented in selected high-incidence regions [15]. This experience is important because it shows how a vaccine can become a population-level preparedness tool when risk geography and target populations are sufficiently defined. However, vaccination is not a stand-alone solution. In Hu County, Shaanxi Province, a free HFRS vaccination programme had been implemented since 1994, yet disease impact remained substantial, and improved vaccination compliance was considered important for control [16]. A recent meta-analysis of inactivated HFRS vaccines supports their protective efficacy in China, while also emphasizing the need to vaccinate populations in infected areas and those at risk of disease [17].

The programmatic implication is that vaccination should be understood as one layer of prevention at defined exposure settings. It is most useful when embedded in surveillance-defined targeting, rodent monitoring, environmental risk reduction, health education and evaluation of coverage and compliance. This also illustrates the danger of overgeneralization. Inactivated HFRS vaccines used in China and other East Asian settings are relevant to Old World HFRS-associated viruses; they should not be presented as ready countermeasures for HCPS-associated viruses such as SNV or ANDV. China’s vaccine experience therefore supports a differentiated countermeasure strategy: match the intervention to the dominant virus, reservoir setting, target population and operational context.

### Climate, land use and urbanization as interface modifiers

The fourth lesson concerns climate, land use and urbanization as modifiers of rodent-human exposure. These factors should not be treated as independent causes of HFRS; rather, they alter the probability that infected rodents, contaminated environments and susceptible humans overlap. A review of urbanization-related environmental factors in China identified population distribution, economic development, land-use change, human activity and vaccination programmes as components of the changing HFRS risk landscape [18]. A national analysis of HFRS under climate variation linked historical HFRS data with climatic and population projections, supporting the view that climate-sensitive changes in reservoir ecology and exposure opportunity may reshape future HFRS risk in China [19]. A 34-year national surveillance analysis further connected HFRS transmission with urbanization, climate, socioeconomic conditions and land-use variables across 31 provincial-level regions [20].

These findings move preparedness beyond static endemic maps. Early warning should incorporate environmental and social change, including cropland, artificial surfaces, wetlands, housing density, agricultural activity and human settlement patterns. At the same time, meteorological effects differ across epidemic regions, and nationwide longitudinal analyses indicate that climate-informed risk assessment must be regionally calibrated rather than applied uniformly [21]. A One Health interpretation is therefore needed: climate and land-use indicators become operationally useful only when linked to local reservoir species, viral circulation and human exposure behaviour [22].

China’s experience therefore defines the preparedness problem for most hantaviruses: persistent reservoir-derived risk can be reduced, mapped and managed, but it should not be assumed to disappear. ANDV raises a different question. What happens when the initial rodent-derived infection does not stop at the environmental exposure setting, but extends into a close-contact human transmission setting? This contrast explains why ANDV should be discussed not as another example of clustering, but as a distinct preparedness problem.

## ANDV as a contrasting interface: when spillover extends to close-contact transmission

ANDV provides a contrasting case to China’s predominantly rodent-derived HFRS experience. Like other HCPS-associated hantaviruses, ANDV can cause severe disease after primary exposure to infected rodent environments. Its distinctive public health significance lies in the additional capacity for secondary human-to-human transmission. The defining feature of ANDV is therefore not simply the occurrence of clustered human disease, because common-source clusters can occur after shared rodent exposure; rather, it is the ability of an initial rodent-derived spillover event to extend through transmission among close human contacts.

Evidence for ANDV person-to-person transmission was first strengthened by Argentine outbreaks in which temporally linked cases, epidemiological investigation and closely related viral sequences supported transmission beyond a common environmental source [23]. Subsequent investigations in Chile identified secondary cases among household members, sexual partners and, less commonly, healthcare contacts [24,25]. A prospective cohort study of 476 household contacts of 76 ANDV cases in Chile found that the risk of HCPS was 17.6% among sexual partners compared with 1.2% among other household contacts, supporting the importance of intimate or prolonged exposure rather than casual household presence [24]. In the 2018–2019 Epuyén outbreak, 34 confirmed cases and 11 deaths were reported, with superspreading-like patterns concentrated within connected social networks rather than diffuse community spread [26]. These patterns are consistent with close interpersonal exposure shaped by host, behavioural and network factors, not unrestricted community transmission.

Recent virological data strengthen the biological plausibility of this pattern. Detection of viral RNA in blood and mucosal or respiratory specimens, and recovery of infectious virus from selected clinical samples, support the possibility of transmission through close exposure to respiratory or oral secretions during acute illness [27]. However, virological detection should not be interpreted as evidence of efficient airborne or casual transmission. Across published outbreaks, secondary transmission has remained primarily associated with intimate contact, caregiving, prolonged indoor proximity, healthcare exposure or unprotected contact with infectious secretions or body fluids.

In the 2026 *MV Hondius* event, WHO reported 13 linked cases, including 12 confirmed and one probable case, and three deaths. The investigation suggested that the initial infection was probably acquired environmentally before boarding, followed by human-to-human transmission onboard. Importantly, completion of 42-day follow-up for identified contacts without additional secondary cases supported effective interruption of transmission and outbreak containment [28]. The event is important not because it overturns the constrained-transmission model of ANDV, but because it shows how limited close-contact transmission can become an international public health challenge when exposed persons and contacts disperse across borders. In contrast to the Yosemite SNV outbreak, where infections were linked to shared exposure in contaminated accommodation, the *MV Hondius* event required both environmental reconstruction and contact-oriented follow-up [28–30].

ANDV therefore requires a dual investigation. Public health teams must identify the initiating rodent-derived exposure and also assess close human contacts who may have been exposed during the infectious period. Risk communication should avoid both extremes: ANDV is not simply another common-source rodent-borne infection, because secondary transmission is documented; but available evidence does not support framing it as an efficiently spreading community respiratory virus. This distinction underpins proportionate preparedness: infection control, contact risk stratification and symptom monitoring are justified for ANDV, whereas such measures should not be generalized to every sporadic hantavirus infection.

## Preparedness tools: shortening the path from exposure to action

In hantavirus preparedness, the central question is not simply whether a diagnostic test, vaccine or treatment exists, but whether available tools can shorten the pathway from exposure to recognition, source reconstruction and proportionate action. A patient with fever and thrombocytopenia after cleaning a rodent-contaminated cabin, a farmer with acute kidney injury after harvest work, a warehouse worker exposed to rats, and a close contact of an ANDV case all require different public health responses. The value of preparedness tools therefore lies in helping clinicians and public health teams decide which interface is involved: shared environmental exposure, rat-associated exposure, or possible ANDV secondary transmission.

Clinical recognition remains the first bottleneck [31]. Early hantavirus illness is non-specific, and the diagnosis is often considered only after renal injury, pulmonary oedema, shock or epidemiological clustering becomes apparent. For HFRS, suspicion should be linked to agricultural exposure, rural housing, crop storage, peri-domestic rat activity and compatible renal or haemorrhagic features. For HCPS, the warning pattern is different: febrile illness followed by thrombocytopenia, haemoconcentration and rapidly progressive cardiopulmonary compromise after exposure to rodent-contaminated buildings, cabins or field settings. Peripheral blood smear review and routine haematological findings can also support rapid presumptive recognition of HCPS when thrombocytopenia, left-shifted granulocytes, immunoblasts and haemoconcentration are present [32]. In suspected ANDV infection, clinical history must extend beyond rodent exposure to include household, intimate, caregiving, healthcare or shared-cabin contact with an ill person. Thus, the exposure history is not an accessory to diagnosis; it is the first step in classifying the event.

Laboratory testing should then answer two operational questions. The first is individual diagnosis: serology remains central, while RT–PCR is particularly useful in early illness, severe disease and outbreak investigation [31,33]. The second is outbreak reconstruction. When multiple cases occur, sequencing can help distinguish repeated environmental acquisition from a linked transmission chain and may identify whether cases share a probable source [34]. This distinction is not academic. A common-source SNV or SEOV cluster requires environmental investigation, rodent control and identification of others exposed to the same setting; an ANDV cluster may additionally require infection-control measures and monitoring of close human contacts. Point-of-care molecular or serological tests and genomic or metagenomic surveillance are therefore most useful when they shorten the time between suspicion and action, rather than when they are presented merely as technological advances [34,35].

Vaccines illustrate the same principle. China’s HFRS vaccination experience shows that immunization can become a public health tool when the dominant viruses, high-risk populations and geographic risk areas are sufficiently defined [15–17]. In this setting, vaccination is one layer of interface-centred prevention, alongside surveillance, rodent control, environmental risk reduction and health education. It is not a universal hantavirus solution. Existing inactivated HFRS vaccines are relevant to selected Old World HFRS settings and should not be extrapolated to SNV- or ANDV-associated HCPS [15–17]. Conversely, ANDV DNA vaccine development is scientifically encouraging, but it remains investigational from a preparedness perspective until protective efficacy, target populations and deployable use cases are established [36].

Treatment and advanced care also need to be framed by timing and syndrome rather than by the word “hantavirus” alone. Early intravenous ribavirin showed benefit in HFRS in China [37], whereas ribavirin did not demonstrate convincing benefit in established North American HCPS [38]. This difference reinforces a broader lesson: once HCPS has progressed to cardiopulmonary failure, preparedness depends less on antiviral availability than on early recognition, rapid transfer and access to advanced supportive care, including extracorporeal membrane oxygenation where feasible [39]. Passive immunotherapy, monoclonal antibodies and drug-repurposing approaches remain important research directions, particularly for ANDV and other HCPS-associated viruses [40–42], but they are not yet substitutes for rapid diagnosis and supportive care pathways.

Preparedness tools should therefore be judged by how well they reduce three delays: delay in suspecting hantavirus disease, delay in determining the exposure or transmission interface, and delay in initiating the correct response. For China-like HFRS settings, this means integrating surveillance, rodent monitoring, vaccination and environmental prevention around defined reservoir settings. For common-source travel clusters, it means reconstructing contaminated places and identifying others who shared that exposure. For ANDV, it means adding close-contact assessment, infection control and symptom monitoring without implying efficient community spread. Table 2 translates these distinctions into decision points for preparedness and response. A tool is useful not because it is modern or technically sophisticated, but because it helps move from a non-specific febrile illness to the right interface-specific action.

**Table 2.** Interface-based decision points for hantavirus preparedness and response.

Scenario	Key question	Evidence to collect	Priority action
Agricultural or rural HFRS	Is this a shared field-rodent exposure?	Farming activities, harvest work, crop storage, rodent signs, seasonality, and other exposed persons	Rodent control, safe cleaning and storage practices, risk education, and targeted vaccination where appropriate
Peri-domestic SEOV	Is there shared rat-associated exposure?	Household or workplace rats, warehouses, ports, food storage, pet-rat or breeding networks, and linked exposed persons	Rat investigation, environmental control, sanitation improvement, and search for others with shared rat exposure
Travel or cabin cluster	Is this a common-source environmental cluster?	Cabin or campsite history, cleaning activities, rodent contamination, ventilation, and shared accommodation history	Environmental assessment, traveller notification, and clinical alerting for those with compatible symptoms
Suspected ANDV	Is there possible secondary human-to-human transmission?	Timing of illness, close contact history, caregiving, shared room or cabin, healthcare exposure, and sequencing where available	Infection control, contact risk stratification, symptom monitoring, and linkage of epidemiological and laboratory findings
Cross-border event	Have exposed persons or contacts crossed geographical borders?	Itinerary, cabin or room assignment, contact lists, travel dates, receiving countries or regions, and available laboratory confirmation	International coordination, information sharing, and risk-based cross-border follow-up

Abbreviations: ANDV, Andes virus; HFRS, haemorrhagic fever with renal syndrome; SEOV, Seoul virus.

## Mobility-aware preparedness beyond cruise travel

Human mobility does not create hantavirus reservoirs, but it can separate exposure, illness recognition, diagnosis and public health follow-up. A traveller exposed in a contaminated cabin, campsite, warehouse or agricultural setting may develop illness after returning home. A military deployment, field expedition or wildlife-tourism trip may involve shared environmental exposure, but cases may be recognized only after the group disperses. SEOV illustrates another mobility-related pattern: infected commensal rats, pet-rat ownership and breeding or trade networks can create linked human cases across households, cities or countries without requiring person-to-person transmission [43]. In these settings, mobility disperses exposed people or infected animals rather than amplifying human transmission.

ANDV adds a second mobility problem because close-contact transmission may occur after the initial rodent-derived infection. In a travel group, ship, remote expedition or shared accommodation, contact intensity, shared indoor air, caregiving and delayed recognition can generate exposures that are difficult to reconstruct once travellers disperse. Preparedness in mobile populations should therefore include mechanisms to preserve itinerary, cabin, household, occupational and close-contact information; identify where environmental exposure may have occurred; arrange medical evaluation and transfer when severe disease develops; and coordinate risk-based follow-up across geographical borders.

Mobility-aware preparedness should be scenario-based. For cabins, campsites and wildlife tourism, advice should emphasize rodent exclusion, safe cleaning, ventilation and avoidance of dry sweeping or vacuuming of contaminated materials. For military and field deployments, pre-deployment education, fever pathways and access to diagnostic testing are essential. For urban rat networks, investigation should focus on shared rat exposure, breeding facilities, pet ownership and supply chains. For suspected ANDV, response must add infection-control precautions, close-contact risk stratification and symptom monitoring. The operational goal is not to treat all hantavirus clusters as transmissible outbreaks, but to rapidly determine whether the event is a common-source environmental cluster, a rat-network exposure, or an ANDV event with potential secondary transmission. Table 2 provides a practical summary of these scenario-based decision points.

## Research and policy agenda

Several priorities follow from an interface-centred and mobility-aware framework. First, endemic countries need integrated human-rodent-virus surveillance systems that combine human case reporting with reservoir monitoring, viral sequencing and environmental data. China’s HFRS experience shows that falling incidence can coexist with persistent viral diversity and shifting affected areas; surveillance should therefore be maintained after apparent control. Second, climate, land-use and urbanization indicators should be linked to local reservoir ecology and human exposure behaviour. Predictive models are most useful when they inform targeted preparedness rather than produce static risk maps.

Continued discovery of novel hantaviruses and expanded recognition of host range also deserve operational interpretation. Viral discovery has shown that hantavirus evolution and host associations are more complex than classical one-virus-one-rodent models suggested [44]. However, a newly detected sequence does not automatically imply a comparable public health threat. The preparedness question is whether a virus is linked to a competent reservoir, shedding or persistence in that host community, human exposure opportunities and compatible disease. Reports linking newly recognized hantaviruses with human HCPS, and recent evidence that SNV may circulate and be shed by taxonomically diverse small mammals, illustrate why discovery should be connected to reservoir ecology and interface-specific exposure assessment rather than treated as a stand-alone warning signal [45,46].

Third, diagnostic capacity should be strengthened for both endemic and travel-associated settings. Rapid serology, RT–PCR and sequencing can support clinical care, distinguish common-source exposure from transmission chains and guide environmental or contact investigations. Fourth, vaccination strategies need a differentiated evidence base. HFRS vaccination programmes should be evaluated as part of integrated control in defined high-risk populations, while next-generation vaccines for HCPS-associated viruses require continued development and efficacy evaluation. Fifth, therapeutic development should remain a priority, including host- or virus-targeted antivirals, drug repurposing, antibody-based interventions and advanced supportive care pathways for severe HCPS.

Finally, public health protocols should clearly separate three scenarios: multiple cases from shared rodent-contaminated environments; linked cases from shared rat networks; and ANDV events with possible secondary human transmission. This distinction is essential for proportionate response. Over-calling every hantavirus cluster as interpersonal transmission may waste resources and create unnecessary alarm, whereas under-recognizing ANDV secondary transmission may delay contact monitoring and cross-border coordination. Figure 1 and Table 2 are intended to make this distinction operational rather than merely conceptual.

## Conclusion

Hantavirus preparedness should be organized around the settings through which human risk is generated. China’s long-term HFRS experience shows that persistent rodent-derived exposure can be reduced but not assumed to disappear; surveillance, vaccination, rodent control, environmental risk reduction and climate- or land-use-informed early warning must remain linked. Its dual HTNV and SEOV ecology demonstrates that a single clinical syndrome can require different prevention strategies depending on the reservoir setting.

ANDV adds a contrasting challenge. In selected settings, an initial rodent-derived infection can extend through limited but consequential transmission among close human contacts, and mobility can transform such events into international follow-up problems. Future preparedness should therefore combine long-term management of rodent-human exposure, rapid discrimination between common-source clusters and ANDV secondary transmission, and response systems that remain effective when exposed persons, infected animals or close contacts cross geographical borders. The next decade should focus on integrated surveillance, rapid diagnostics and sequencing, risk-defined vaccination, targeted therapeutic development and operational protocols that are specific enough to distinguish shared exposure from transmission, yet flexible enough to work when people move.
